# Renal Cell Carcinoma With Hemangioblastoma‐Like Features, Fibromyomatous Stroma, and TSC1 Mutation

**DOI:** 10.1155/carm/3548941

**Published:** 2026-08-23

**Authors:** Shiyun Yao, Jinghui Huang, Mingfa Wang, Chunji Quan

**Affiliations:** ^1^ The First School of Clinical Medicine, Hainan Medical University, Haikou, Hainan, China, hainmc.edu.cn; ^2^ Department of Pathology, The Second Affiliated Hospital of Hainan Medical University, Haikou, Hainan, China, hainmc.edu.cn; ^3^ Department of Radiology, The Second Affiliated Hospital of Hainan Medical University, Haikou, Hainan, China, hainmc.edu.cn; ^4^ Department of Pathology, Hainan Women and Children’s Medical Center, Haikou, Hainan, China

**Keywords:** fibromyomatous stroma, hemangioblastoma, renal cell carcinoma

## Abstract

Renal cell carcinoma (RCC) with fibromyomatous stroma (RCC‐FMS) was classified as an “emerging/provisional” entity in the 2016 WHO classification of tumors, specifically categorized as RCC with (vascular) fibromyomatous stroma. However, it was not included in the 2022 WHO classification. Renal cell carcinoma with hemangioblastoma (RCC‐HB)‐like features has also been reported infrequently in recent years. RCC‐HB‐like features and fibromyomatous stroma is quite rare, with only two reports in the literature. This report describes a rare tumor of RCC‐HB‐like features and fibromyomatous stroma associated with TSC1 mutations. A female patient was incidentally discovered a right renal mass during a routine physical examination conducted 2 weeks prior. Histology revealed that the tumor consisted of three distinct components: clear cell papillary renal cell tumor (CCPRCT)‐like areas, fibromyomatous stroma, and HB‐like areas. Immunohistochemical staining showed that the CCPRCT‐like components exhibited basolateral, cup‐shaped CAIX staining and were diffusely positive for AE1/AE3, CK7, and PAX‐8, partially positive for AMACR/P504S, and weakly positive for CD10. The fibromyomatous stroma was positive for H‐caldesmon, desmin, and SMA. In the HB‐like components, α‐inhibin, ERG, CD34, CAIX, and vimentin were all diffusely positive, CD10 was partially positive, and PAX‐8 showed scattered, weak positivity. Whether RCC‐HB features and fibromyomatous stroma can be regarded as a distinct subtype of RCC remains to be discussed by accumulation of more cases.

## 1. Background

Hemangioblastoma (HB) is a benign tumor, which usually occurs in the central nervous system. In the 2016 WHO classification of renal tumors, renal HB is a new subtype of mesenchymal tumors, and its biological behavior is benign. In recent years, renal cell carcinoma with hemangioblastoma (RCC‐HB) has been successively reported. Renal cell carcinoma with fibromyomatous stroma (RCC‐FMS) is a tentative subtype in the 2016 WHO classification of epithelial tumors of the kidney. However, it was not retained in the 2022 edition. In contrast, the 2022 WHO classification introduced a novel entity, *ELOC (TCEB1)-*mutated renal cell carcinoma (RCC), which is morphologically characterized by prominent leiomyomatous stroma and also harbors *ELOC (TCEB1)* gene mutations.

## 2. Rarity

RCC‐HB‐like features and fibromyomatous stroma are extremely rare, with only two reports in the literature, one of which was accompanied by special molecular changes: mutations in tuberous sclerosis complex (TSC)2 and SETD2 [1]. In this paper, we report a rare renal tumor with TSC1 mutation. Given the unique morphologic features of this tumor, it may represent a new tumor or a variant of RCC‐FMS.

### 2.1. Case Summary

**FIGURE 1:**
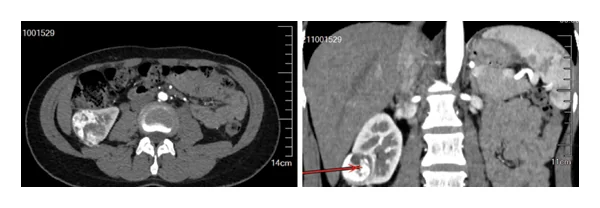
Enhanced CT features of the tumor. A slightly hypodense mass is observed at the lower pole of the right kidney. The mass exhibits heterogeneous density with indistinct margins, with a size of 42 × 30 × 33 mm, partially protrudes beyond the renal contour. The tumor demonstrates marked heterogeneous enhancement during the arterial phase on contrast‐enhanced scan. The enhancement effect diminishes during the venous and delayed phases. Cystic density is observed within the lesion.

A 36‐year‐old female patient was referred for evaluation after a right renal mass was incidentally discovered during a routine physical examination conducted 2 weeks prior. B‐ultrasound revealed an isoechoic solid mass located in the lower pole parenchyma of the right kidney, measuring approximately 38 × 35 mm in size, with an ill‐defined margin, suggestive of a neoplastic process. Contrast‐enhanced CT of the kidney demonstrated a slightly hypodense mass at the lower pole of the right kidney with an ill‐defined border, measuring approximately 42 × 30 × 33 mm in size, with partial extension beyond the renal contour (Figure 1).

Gross examination of the resected specimen revealed a gray–red, partially encapsulated nodule measuring 3.5 × 2.5 × 2.5 cm in size. Histopathological analysis showed that the tumor was composed of three distinct components: a clear cell papillary renal cell tumor (CCPRCT)‐like component, a HB‐like component, and a fibromyomatous component.

### 2.2. Morphology

**FIGURE 2:**
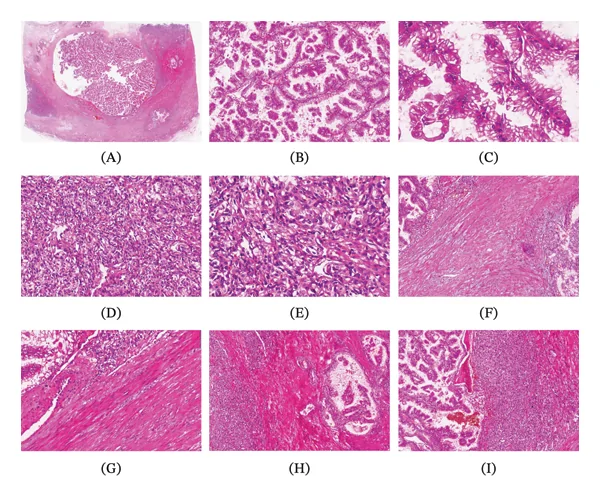
(A) Histological examination under low magnification reveals that the tumor comprised three distinct components. (B) The tumor cells of CCPRCT demonstrate papillary proliferation, accompanied by the presence of fibrovascular cores. (C) The cytoplasm exhibits a clear to eosinophilic appearance, with nuclei that are moderately enlarged and contain discernible nucleoli. (D) The tumor cells of the hemangioblastoma‐like component form an arborizing thin‐walled of capillary vessels. (E) The tumor cells were polygonal, and part of the cytoplasm was rich in lipid vacuoles. (F) and (G) The proliferating spindle cells were arranged in bundles, and the cytoplasm was rich in eosinophilus, showing fibromyomatous appearance. (H) and (I) The three tumor components cross each other.

CCPRCT‐like component: The tumor cells showed papillary hyperplasia, with visible fibrous vascular axes, featuring clear or eosinophilic cytoplasm, medium‐sized nuclei, and visible nucleoli. HB‐like component: The tumor cells were arranged in a thin capillary network, displaying polygonal morphology, with some cells containing abundant lipid vacuoles in their cytoplasm. Between the above two components, proliferative spindle cells were observed arranged in fascicles, exhibiting abundant eosinophilic cytoplasm, resembling leiomyoma (Figure 2).

### 2.3. IHC

**FIGURE 3:**
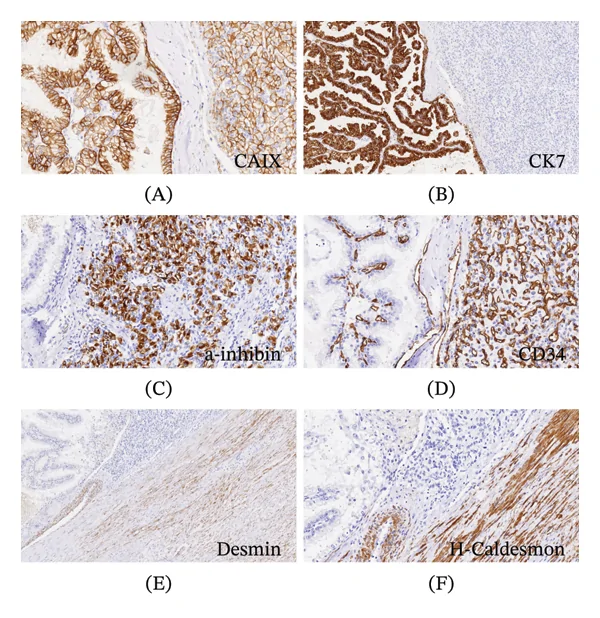
Immunohistochemical staining. (A) CAIX in CCPRCT‐like region was cup positive, while in HBs‐like region was diffusely positive. (B) CK7 in CCPRCT‐like region was diffusely strong positive and in HBs‐like region was negative. (C) a‐inhibin in HBs‐like region was diffusely strong positive. (D) CD34 in HBs‐like region was diffusely positive. (E) Desmin was diffusely positive in the fibromyomatous stroma. (F) H‐Caldesmon was diffusely strong positive in the fibromyomatous stroma.

Immunohistochemical analysis revealed distinct staining patterns across different tumor components. In the CCPRCT‐like component, tumor cells demonstrated positivity for AE1/AE3, CK7, CK34βE12, EMA, PAX‐8, vimentin, and FH, with CAIX showing characteristic basolateral, cup‐shaped positivity. AMACR/P504S and CD10 exhibited weak immunoreactivity. The HB‐like component showed diffuse positivity for α‐inhibin, ERG, CD34, CAIX, vimentin, and FH, with CD10 demonstrating focal positivity and PAX‐8 showing scattered and weak positivity. In the fibromyomatous component, smooth muscle markers including H‐caldesmon, desmin, and SMA were uniformly positive (Figure 3). Next‐generation sequencing (NGS) identified a pathogenic *TSC1* p.N837fs inactivating mutation. The patient’s postoperative course was unremarkable, and they were discharged without additional treatment. At the 6‐month postoperative follow‐up, the patient remained asymptomatic with no evidence of recurrence or metastatic disease.

## 3. Discussion

HBs are benign tumors that predominantly occur in the central nervous system, some of which are associated with von Hippel–Lindau (VHL) disease, while others are discovered incidentally. There is also increasing evidence of sporadic HBs occurring outside the central nervous system [2]. In the 2016 WHO classification of renal tumors, renal HB was recognized as a new subtype of mesenchymal tumors. At low magnification, the tumor cells exhibited slightly lobulated architecture with dendritic thin‐walled vessels and matrix hyalinization. At high magnification, the tumor cells appeared oval or polygonal, displaying abundant eosinophilic or clear cytoplasm, lipid vacuoles, and eosinophilic hyaline globules. Immunohistochemical staining demonstrated that the tumor cells were positive for a‐inhibin, S‐100, PAX‐8, NSE, and vimentin [3]. Renal HB is commonly associated with VHL disease; however, recently, Raja F et al. reported a case of sporadic renal HB in a patient without a history of VHL [4].

In recent years, a limited number of cases featuring clear cell RCC‐HB‐like characteristics have been documented. Among these cases, the RCC components consistently manifest as clear cell renal cell carcinoma (CCRCC) with varying proportions, predominantly characterized by HBs. In the case reported by Rodolfo et al., a distinct transition zone between CCRCC and HBs was observed [5]. Conversely, Sankalp et al.’s case demonstrated no such transition zone between the two components [6]. The majority of reported cases reveal that the tumor is primarily composed of HBs, with the CCRCC component only identified through extensive sampling and thorough dissection. This observation underscores the necessity for caution when diagnosing simple renal HB. RCC‐HBs exhibit no unique molecular alterations. The classification of RCC‐HBs as an independent tumor type or a distinct subtype of CCRCC warrants further discussion and analysis through additional case studies.

RCC‐FMS was classified as an “emerging/provisional” entity in the 2016 WHO classification of tumors, specifically categorized as RCC with (vascular) fibromyomatous stroma. This tumor exhibits significant histological overlap with CCRCC and CCPRCT [6]. From a molecular genetic perspective, RCC‐FMS demonstrates various molecular abnormalities, including mutations in *TSC1, TSC2, MTOR*, and *ELOC (TCEB1)*, while lacking the chromosome 3p deletion and *VHL* mutation that are typically observed in CCRCC [7]. In the 2022 WHO classification of tumors of the urinary system and male reproductive organs, only *ELOC* (formerly *TECB1)*‐mutated RCC (*ELOC*‐mutated RCC) were separated from RCC‐FMS and established as a distinct subtype. This subtype exhibits tumor cells with clear cytoplasm and distinct cell borders, and is morphologically defined by prominent leiomyomatous stroma, with the *ELOC (TCEB1*) gene mutation at 8q21.11 as its characteristic molecular alteration. However, RCC‐FMS cases with alterations in the MTOR/TSC pathway have not yet been recognized as an independent subtype [8].

However, Melissa Tjota et al. analyzed 500 cases of CCRCC in the United States, identifying 12 cases with prominent smooth muscle stroma. Through NGS, they detected mutations in *TSC1*, *TSC2*, and *MTOR* in all these cases, with one case exhibiting TSC1 deletion. Despite the limited sample size, Tjota et al. proposed that RCC with leiomyoma stroma associated with *TSC/MTOR* mutation represents a distinct tumor entity [9].

RCC‐HB‐like features and fibromyomatous stroma are exceptionally uncommon, with only two cases reported in the literature. In one of the cases, the tumor was predominantly composed of renal HB, while a minor component demonstrated morphological features histologically consistent with CCPRCT. Transition areas and merging between the two components were observed. Smooth muscle bundles were seen interspersed within the tumor parenchyma [10]. In the other case, the tumor was predominantly composed of RCC morphologically characterized as CCRCC, with intersecting and transitional areas between the carcinoma and HB components. This case represents the first reported instance of RCC‐HB‐like features and fibromyomatous stroma, harboring mutations in *TSC2* and *SETD2* [1].

Several retrospective reports published in recent years have described a small number of RCC cases exhibiting HB‐like features and fibromyomatous stroma. Some of these cases arose in patients with TSC, whereas others occurred in a sporadic setting. The median age at presentation ranged from 21 to 55 years, and the mean tumor diameter was approximately 3 cm. All tumors presented as well‐circumscribed solid nodules, with relatively consistent immunohistochemical profiles across their various histological components. NGS revealed mutations or deletions in *TSC1*, *TSC2*, or *MTOR*, and long‐term follow‐up demonstrated no evidence of tumor progression (Table 1).

**TABLE 1:** Compilation of articles related to RCC‐FMS or RCC‐HB‐FMS.

	Case 1	Case 2	Case 3	Case 4	Case 5	Case 6	Case 7	This case
Author, Year	Chen et al. 2020 [10]	Kong et al. 2022 [1]	Shah et al. 2020 [11]	Tjota et al. 2023 [9]	Baranova et al. 2025 [12]	Trpkov et al. 2026 [13]	Tao et al. 2026 [14]	Current study

Cases	1	1	18	12	3	3	7	1

TSC patients	NO	NO	NO	NO	YES	2 NO, 1 YES	NO	NO

Age/sex	41/Female	36/Female	Mean age 52/M:F = 1:2	Mean age 52/M:F = 7:5	Mean age 21/Female	Mean age 43/M:F = 2:1	Mean age 55/M:F = 5:2	36/Female

Tumor size	2.3 cm	2 cm	Average 2.3 cm	Average 2.5 cm	average 2.7 cm	Average 2.8 cm	Average 2.5 cm	3.5 cm

Gross features	Solid, relatively well‐circumscribed	Solid, relatively well‐circumscribed	Solitary, solid circumscribed	Solitary/multiple, circumscribed, solid	multiple, circumscribed	Circumscribed, solid	Circumscribed, solid	Solid, partially encapsulated

Histological features	CCPRCT + HB + FMS	CCRCC + HB + FMS	CCRCC + FMS	CCRCC + FMS	CCRCC + HB + FMS	CCRCC + HB + FMS	CCRCC/CCPRCT + HB + FMS	CCPRCT + HB + FMS

IHC	CCPRCT: CK7+, CAIX+(cup shaped) HB: α‐inhibin+, ERG+, CD34+ FMS: H‐caldesmon+, Desmin+	CCRCC: CK7+, CAIX + HB: α‐inhibin +, CD34+ FMS: SMA+	CCRCC: CK7+, CAIX + FMS: Desmin+	CCRCC: CK7+,CAIX+	CCRCC: CK7+, CAIX + HB: α‐inhibin+, CD34+ FMS: SMA+, Desmin+	CCRCC: CK7+, CAIX + HB: α‐inhibin +, CD34+	CCRCC: CK7+, CAIX+ CCPRCT: CK7+,CAIX+(cup‐shaped) HB: α‐inhibin +, CD34+ FMS: SMA+, H‐caldesmon+	CCPRCT: CK7+,CAIX+(cup‐shaped) HB: α‐inhibin+, ERG+, CD34+ FMS: H‐caldesmon+, Desmin+

Gene	None	TSC2 and SETD2	TSC1 (4), TSC2 (4), MTOR (6), and/or ELOC (2)	TSC1 (5), TSC2 (3), MTOR(4)	TSC1	TSC1 (1), TSC2 (2)	TSC1 (2), TSC2 (2), MTOR (3), NBPF1 (1)	TSC1

Follow‐up	4‐month, no recurrence or metastasis	18‐month, no recurrence or metastasis	median follow‐up of 48 months, no recurrence or metastasis	median follow‐up of 43 months, no recurrence or metastasis	follow‐up (10 months‐21 years), no recurrence or metastasis	6 months–10 months, no recurrence or metastasis	median follow‐up of 46 months, no recurrence or metastasis	6‐month, no recurrence or metastasis metastatic

*Note:* HB, hemangioblastoma; FMS, fibromyomatous.

Abbreviations: CCPRCT, clear cell papillary renal cell tumor; CCRCC, clear cell renal cell carcinoma; TSC, tuberous sclerosis complex.

**FIGURE 4:**
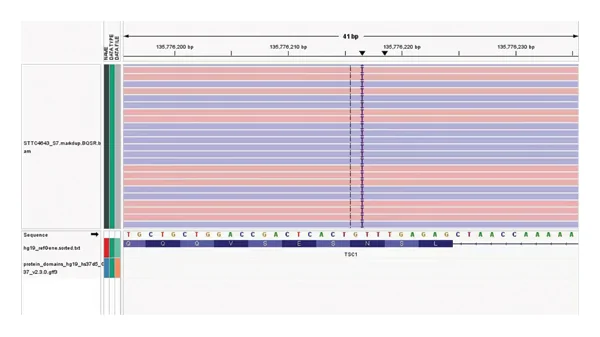
An inactivating mutation in the TSC1 gene (nucleotide change c.2510dup, amino acid change p.N837fs) was detected by next‐generation sequencing (NGS).

The CCPRCT component accounted for approximately 60% of the cases reported in this study. Morphologically, these tumors present as atypical clear cell papillary renal cell tumors that are well circumscribed and exhibit true papillae with clear cytoplasm, while showing inconspicuous nuclear displacement away from the basement membrane. The immunohistochemical profile is characterized by typical cup‐shaped CAIX positivity, along with CK7 positivity and focal positivity for P504S and CD10. Approximately 30% of the tumor consisted of HB areas, which showed positive immunohistochemical staining for α‐inhibin, ERG, CD34, and CAIX. Focal transitional zones between these areas and the CCPRCT component were also observed. The fibromyomatous component demonstrated positive staining for smooth muscle makers, including H‐caldesmon, desmin, and SMA. Each tumor component exhibited its own distinct morphology and immunohistochemical expression, with clear transitional zones between them. NGS of the tumor sample revealed an inactivating TSC1 p.N837fs mutation, which represents the first documented occurrence of this genetic alteration in such cases (Figure 4).

Compared with these previously reported cases, the present tumor showed concordant morphological composition and immunohistochemical characteristics among its constituent elements. NGS of the tumor sample identified an inactivating frameshift mutation, TSC1 p.N837fs. Collectively, these findings suggest that, although rare, this group of neoplasms exhibits a high degree of histomorphological and immunohistochemical consistency. In previous diagnostic practice, pathologists may have focused predominantly on the RCC component while overlooking the other two benign elements, potentially leading to misdiagnosis or underdiagnosis.

TSC is an autosomal dominant genetic disorder. The condition manifests three primary renal lesions: angiomyolipomas, cysts, and RCC. Patients with TSC‐associated renal tumors typically present at an average age of 36 years, showing a female predominance with a ratio of 2:1. In sporadic RCC cases, both RCC‐FMS and eosinophilic cystic renal cell carcinoma (ESC‐RCC) demonstrate relatively high mutation rates in TSC1 and TSC2 genes [15].

Whether RCC‐HB‐like features and fibromyomatous stroma represent a distinct subtype remains uncertain. Although molecular genetic overlap with RCC‐FMS has been observed, the classification as a tumor variant of RCC‐FMS requires the accumulation of additional cases for comprehensive analysis.

In the 2022 WHO Classification of Tumours of the Urinary System and Male Genital Organs, “clear cell papillary renal cell carcinoma” was reclassified as “clear cell papillary renal cell tumor” (ICD‐O code: 8323/1). It is an indolent epithelial tumor, which can also exhibit fibromyomatous stroma. Therefore, it is sometimes difficult to distinguish it morphologically from RCC with fibroma‐like or fibromyomatous stroma (RCC‐FMS) or *ELOC*‐mutated RCC, both of which often contain mutations in *TSC1*, *TSC2*, *MTOR*, or *ELOC (TCEB1)*. However, the 2022 WHO classification no longer emphasizes RCC‐FMS as a distinct entity and has introduced a new tumor type: ELOC (formerly *TCEB1*)‐mutated RCC. The most prominent morphological feature of this tumor is a smooth‐muscle‐rich stroma. The tumor cells predominantly exhibit acinar or papillary growth patterns, with clear cytoplasm and well‐defined cell borders. Immunohistochemically, the tumor cells express CAIX, CK7, and CD10. A characteristic molecular alteration is mutation of the ELOC (*TCEB1*) gene, located at 8q21.11. Therefore, when a tumor demonstrates clear cytoplasm, predominantly papillary or acinar architecture, and a fibromyomatous stroma, molecular testing is recommended to further confirm the diagnosis.

As a single case report, this study has limitations. First, the single‐case design limits generalizability, so our findings must be interpreted cautiously. Second, the 6‐month follow‐up is too short to evaluate late recurrence risk; longer surveillance is needed. Third, because few comparable studies exist (mostly retrospective reports), our literature search may have missed some articles, and publication bias cannot be ruled out, potentially affecting the objectivity of our comparisons. Larger case series and longer follow‐up are required to confirm our observations.

## Funding

The authors have nothing to report.

## Conflicts of Interest

The authors declare no conflicts of interest.

## Data Availability

The data that support the findings of this study are available from the corresponding authors upon reasonable request.
